# What about the Consequences of the Use of Distance Learning during the COVID-19 Pandemic? A Survey on the Psychological Effects in Both Children and Parents

**DOI:** 10.3390/ijerph182312641

**Published:** 2021-11-30

**Authors:** Maria Grazia Maggio, Maria Chiara Stagnitti, Patrizia Calatozzo, Antonino Cannavò, Daniele Bruschetta, Marilena Foti Cuzzola, Alfredo Manuli, Giovanni Pioggia, Rocco Salvatore Calabrò

**Affiliations:** 1 Department of Biomedical and Biotechnological Science, The University of Catania, 95123 Catania, Italy; mariagraziamay@gmail.com; 2 Studio di Psicoterapia Relazionale e Riabilitazione Cognitiva, 98124 Messina, Italy; mariachiara.stagnitti@gmail.com (M.C.S.); patrizia.calatozzo@gmail.com (P.C.); marilenafoti@yahoo.it (M.F.C.); 3 AOU Policlinico “G. Martino”, 98125 Messina, Italy; antonino.cannavo.85@gmail.com (A.C.); d.bruschetta@unime.it (D.B.); manulialfredo@gmail.com (A.M.); 4 Institute for Biomedical Research and Innovation, National Research Council of Italy (IRIB-CNR), 98164 Messina, Italy; giovanni.pioggia@irib.cnr.it; 5 IRCCS Centro Neurolesi “Bonino Pulejo”, 98121 Messina, Italy

**Keywords:** COVID-19, distance learning, psycho-emotional impact

## Abstract

Background: The COVID-19 pandemic implicated many social restrictions, including the use of distance learning (DL). Indeed, parents were obligated to support their children in online lessons and schoolwork. The aim of this study was to investigate the psycho-emotional impact of the COVID-19 pandemic on parents and children submitted to DL. Methods: One hundred and ninety-two participants (96 parents and 96 children) were enrolled in this study. Parents and children completed an online questionnaire, structured in four sections. Results: The results showed that parents had higher levels of stress and anxiety. In particular, the stress for DL was positively correlated with depression and anxiety. Parents’ jobs were negatively correlated with their levels of anxiety and stress. On the other hand, children reported higher levels of depressive symptoms and event-related anxiety, which increased as children got older. The stress and the anxiety in parents were positively correlated with the mood depression and anxiety of their children. Conclusions: The COVID-19 pandemic had a negative impact on the psychological well-being of children and parents who used DL. Although DL could be an alternative teaching method during pandemics, face-to-face teaching is fundamental and irreplaceable as it encourages dialogue, involvement, and human contact.

## 1. Introduction

The COVID-19 pandemic, an infectious disease caused by the new coronavirus SARS-CoV-2, is a global health problem that has affected millions of people since January 2020 [1]. To limit COVID-19 transmission, national governments took precautionary actions, such as adopting careful personal hygiene, wearing masks and gloves, and implementing social distancing [2]. In particular, the Italian government adopted measures aimed at limiting social contacts, including the closure of public places (i.e., schools, offices, theatres, restaurants, bars, parts of public transport) and exhorting people to stay at home [3]. These measures of social distancing caused substantial changes in daily social life, affecting children’s, adolescents’, and parents’ lifestyles. The use of smart-working and distance learning (DL) forced children and parents to spend a lot of time at home, in front of their computer and smartphone screens [3,4]. Moreover, the COVID-19 pandemic caused a socio-economic crisis with job losses, financial insecurity, mental health problems, and lack of care services, including childcare services [5]. All these stressors had negative effects on the mental well-being of each family member [3]. In more detail, about one in four parents reported worsening mental health, and one in seven parents had worsening behavioral health for their children since the pandemic began. Of note, the worsening of parental mental health and children’s behavioral health were at times intertwined, with nearly 1 in 10 families reporting worsening of both. This resulted in loss of childcare, delays in health care visits, and worsened food security.

The United Nations Educational, Scientific, and Cultural Organization estimated that during the pandemic, 1.38 billion children have been out of school or childcare, without access to group activities, team sports, or playgrounds [6]. The closing of schools and lack of childcare services obligated parents to take greater responsibilities for their children’s care and home education, supporting them during distance learning (DL) with online lessons and schoolwork [7,8,9], leading to negative impacts on the well-being of children and parents [4,5,7,8,10,11]. In more detail, it has been shown that the negative impact of the pandemic depends on how parents are able to adapt. According to Fegert et al., more flexible parents tend to see pandemic limitations in a positive way, due to the opportunity to spend more time in the family; on the other hand, for others, the pandemic represents a threat to family well-being and personnel, eliciting unresolved conflicts [12]. Indeed, there has been an increase in family violence, child abuse, and neglect during the pandemic [5,7,8,9,13]. What is more, parental emotional and physical burnout can be linked to other factors, such as the type of daily activities that involve children, and chronic and critical stresses, such as the presence of diseases [4,5,7]. Excessive parental exhaustion could cause a sense of fatigue in parenting activities with little emotional involvement and/or estrangement from children, which significantly affects the mental well-being of children [5,14]. Additionally, a survey found that another concern for parents is their children’s mental and emotional health. Some authors have shown that closing school and home daily routines can be harmful to children, especially if they have a behavioral disorder [8,9,15]. Furthermore, children’s cognitive and emotional regulation systems are immature and can be vulnerable to the psychological effects of the pandemic with negative outcomes. Some recent studies have found higher rates of anxiety and depression in children than adults [8,9,10,11,16]. DL could allow the maintenance of school routines and contact with peers, offering parents a chance to receive help from teachers. It uses technology to enable students to learn without being physically present in the classroom, thus individualizing the learning process [17]. However, few studies have investigated the psychological effects of the pandemic on parents and children while also considering the effect of DL.

Thus, the present study sought to investigate whether and to what extent the use of DL during the COVID-19 pandemic has affected the psychological well-being of a sample of Italian children and their parents.

## 2. Materials and Methods

### 2.1. Participants and Settings

We used a cross-sectional survey design to assess the psychological effects of the lockdown due to the COVID-19 pandemic on both parents and children, using an anonymous online questionnaire. The online survey was administered using some common tools found on smartphones (e.g., WhatsApp, Facebook) or e-mail. Potential study participants were identified through school records of the main primary and secondary schools in the province of Messina, Sicily, after a previous contact with the principal, who informed teachers and parents about our research.

Inclusion criteria for parents were: (i) to live in Messina, and (ii) to be the main person responsible for the DL of the children. To be included in the study, children had to attend compulsory education with an age range of 5–16 years.

The final sample consisted of 96 parents (94.8% women; mean age in years 44.62 ± 5.30) and 96 children (51% females; mean age in years 11.81 ± 3.23).

### 2.2. Procedures

Participants were interviewed online, as it was not possible to administer tests using face-to-face modalities, due to the restrictive measures of the COVID-19 pandemic. They filled out the questionnaires in Italian, through an online survey platform, reached by a simple link (Table 1). The data collection was performed from 17 March 2020 to 2 May 2021.

This study complies with the principles contained in the Declaration of Helsinki and all participants provided informed consent. Anonymity was guaranteed by the online form, in which the data were password-protected and managed only by those responsible for the research.

### 2.3. Outcome Measures

The survey consisted of several sections. The first part consisted of a structured interview on the socio-demographic data (gender, age, education, schooling, city of birth, profession) of both the caregiver and child/student who used DL. The second part presented a series of psychological scales for assessing the psychological impact of DL on parents. 

The psychological battery included: 

- The Depression Anxiety Stress Scales (DASS-21), a questionnaire validated on the Italian population and composed of 21 items on a 4-point Likert scale that measures anxiety, stress, and depression. Regarding internal consistency, Cronbach’s alpha coefficient is 0.87 for depression, 0.80 for anxiety, 0.89 for stress [18,19].

- The Stress for Distance learning in the COVID-19 era (SDC-Q) is a questionnaire of 6 questions on a 4-point Likert scale. This questionnaire examines the perception of stress in the family’s management caused by the use of DL. 

The third part included tests that children and adolescents had to fill in via self-assessment with parental support: 

- The Children Depression Inventory (CDI) is a self-assessment scale validated on the Italian population for depression that can be used with children between the ages of 8 and 17. The test consists of 27 items; each item has three possible answers with a score from 0 to 2. The psychometric characteristics of CDI have been reported in many studies. Researchers typically report internal coherence reliability coefficients around 0.80 [20,21]; and test-retest reliability coefficients ranging around 0.87 [21,22].

- The State-Trait Anxiety Inventory for Children (S.T.A.I.C.) is a tool for measuring anxiety with upper-elementary- or junior-high-school-aged children and consists of two twenty-item scales. The Cronbach’s alpha coefficient of the STAI is 0.82 [23,24].

Finally, both groups were administered the System Usability Scale (SUS) to evaluate the usability of DL. The system usability scale (SUS) is a Likert scale with ten items that provides a global view of subjective usability assessments. SUS requires only one evaluation at the end of the treatment; scores above 50.0 indicate good usability of the device [25].

### 2.4. Statistical Analysis

Statistical analysis was performed using SPSS Statistic 16.0 (IBM SPSS Statistics, New York, NY, USA). The descriptive statistics were analyzed and expressed as mean ± standard deviation or as median ± first third quartile for continuous variables, as appropriate; frequencies (%) were used for categorical variables. Clinical scale scores were expressed as a mean and standard deviation; the perception of usability of the questionnaire was expressed in percentages. We used linear regressions to calculate the univariate associations between two categorical variables. All tests were two-tailed, with a significance level of *p* < 0.05.

## 3. Results

One hundred and ninety-two participants (96 parents: mean age ± SD: 44.62 ± 5.30 years; and 96 children: mean age ± SD: 11.81 ± 3.23) were included in the study. A more detailed description of the sample is reported in Table 1. All of the participants completed the online questionnaire, with good usability of the tool and without reporting excessive difficulties. In fact, both parents (89%) and children (94%) indicated that the questionnaire was easy to fill out.

As shown in Table 2, parents had higher levels of stress (SDC Questionnaire: 4.41 ± 4.1; DASS-21 S: 10.66 ± 4.3) and anxiety (DASS-21 A: 9.03 ± 4.1). The children reported higher mood depression (CDI: 24.04 ± 3.3) and anxiety (S.T.A.I.C. 1: 41.81 ± 4.9). 

Following linear regression analysis, there were no statistically significant differences in sex and age of the children, or in levels of anxiety (*p* = 0.621), stress (*p* = 0.116), depression (*p* = 0.756), or stress for DL (*p* = 0.324) in the parent group.

Parental stress was positively correlated with anxiety (*p* < 0.001), depressive symptoms (*p* < 0.001), and stress for DL (*p* = 0.004), whereas in children/adolescents, depression correlated with their anxious status (*p* < 0.001 for both tests). Furthermore, as the children got older, their depressive and anxious symptoms increased.

Parental depression symptoms were correlated with their children’s anxiety symptoms (*p* = 0.03 for both tests). Moreover, parents’ anxiety and stress were correlated with anxiety and depressive symptoms of their children (*p* < 0.01 for both tests). The stress for DL during COVID-19 in parents was associated with trait anxiety of their children/adolescents (*p* < 0.001). 

Children’s depression symptoms correlated with their parent’s anxiety levels (*p* < 0.001) and stress (*p* = 0.01). A higher level of anxiety in children/adolescents was also positively correlated with parental stress (*p* = 0.003).

Finally, we observed that 61.5% of children/adolescents liked to use the DL system and would like to employ it in the future (54.2%), but they believed that this system was not the same as face-to-face lessons (60.4%). 

As regards acceptance of the DL, we found high usability, as parents obtained an average score of 70.47 (SD 19.9) and children of 70.46 (SD 19.9).

## 4. Discussion

In our study, we evaluated the psychological impact of COVID-19 on Italian children aged 5–16 and their parents, with regard to the use of DL. Our results showed that lockdown measures due to the COVID-19 have negatively affected the behavioral and emotional aspects of both children and parents. Regarding the well-being of children during the quarantine, our results underlined high levels of depressive symptoms and event-related anxiety, compared to the general population. This finding is in line with previous reports, which highlight an increase in emotional symptoms in children during periods of lockdown [6,9]. Moreover, our sample showed high levels of parents’ anxiety and stress, as compared to the general population. The data are in line with previous studies [7,8,9,10], which have also been carried out in other countries [26,27]. Finally, the parental symptoms are related to the psychological symptoms of the children. Given that the presence of higher levels of stress and the onset of psychological problems in parents can adversely affect the psychological well-being of children [10,11,12,13,14,15,16], cooperation between parents and teachers is essential not only for educational purposes but also in the support of children [9,10]. For this reason, DL could be a good education tool in the lockdown phase, also considering that most of the children/adolescents liked the DL system and would like to use it in the future. Furthermore, both children and parents have declared high usability and acceptance of the DL.

To the best of our knowledge, few studies have been carried out to evaluate well-being in the parent–child dyad [5,7,8,9,10,28,29] during the pandemic, especially investigating the correlation between emotional symptoms (anxiety, depression, stress) of parents and children. However, the COVID-19 pandemic has significantly affected family daily life by changing established routines and presenting new educational challenges for parents and children. In particular, the restrictions on going out and the need to use DL have forced parents to spend many hours managing their children and the psychological problems deriving from the reduction of social activities. Parents and children faced an unknown situation with a highly stressful value, amplified by the media hype and by the uncertainty and fear of the virus [6]. Orgilés et al. [10] observed that 85.7% of parents perceived changes in their children’s emotional state and behaviors during the quarantine. Moreover, parents had higher levels of anxiety and psychological distress, and lower levels of perceived self-control and psychological well-being [4,5,7,8,11]. In particular, the risk of psychological distress is higher in parents of children with pre-existing psychological and behavioral difficulties, who require personalized teaching because of their special needs [4,7,30]. This aspect is particularly important in DL, where the presence of remote teaching can reduce the active involvement of the child, who also needs the parent to solve the technical problems of the DL platforms [31]. This is why parents of children with intellectual disabilities present with a higher burden and stress. 

Notably, in our study, nearly all parents responding to the questionnaire were females. These data are in line with the literature [7], demonstrating that the pandemic had a negative psychological impact on Italian mothers, who are mainly responsible for the child’s management, especially regarding DL. Indeed, it has been shown that quarantine and COVID-19 restrictions can be perceived as an uncertain and threatening situation, capable of triggering symptoms of anxiety [32] and stress [33]. 

Furthermore, as observed by Cusinato et al. [7], our study shows that some socio-demographic and contextual variables can influence parental well-being. Particularly, the type of profession, such as freelancer or unemployment, is related to greater stress and anxiety. This is probably due to the economic uncertainties of these jobs that prevent them from finding the right strategies to deal with it. 

According to our results, there is a positive association between the emotional symptoms of the parents (in particular the DL-related stress experienced in the COVID-19 period) and the depressive and anxious symptoms of the children. This further supports the idea that, in the parent–child dyad, there is a reciprocal enhancement of negative symptoms, which can affect the quality of life. Moreover, these data underline the mutual influence between the psychological health of children and parents [7].

It is, therefore, essential that both parents and children are considered in planning interventions in the family environment, avoiding isolated approaches. In fact, it has been shown that parents who have a better psychological adaptation can experience fewer difficulties in their parental role, and this in turn can positively affect the well-being of their children [7]. To this aim, the family should be considered from a systemic perspective, in which all family members mutually influence each other’s adaptation, favoring the development of new resources that promote well-being even in difficult times, including pandemics [1,34]. This important approach may be of help when dealing with DL, which has revolutionized the way children learn and study, involving parents more than in previous times. 

We believe that it is important to deepen these issues, as remote school and teleconferencing could be a useful resource when integrated with normal teaching, also considering their impact on the family context [1].

Another important result of our study is that we did not find statistically significant relationships between the child’s age and sex and the levels of anxiety, stress, depression, and stress of their parents. However, it would be useful to explore this aspect in larger samples involving more male parents to confirm these results.

Despite the interesting results, our study has some limitations. First of all is the online modality to assess the participants. In fact, although it allowed us to reach a fair sample during the lockdown, it did not allow us to control for some contextual variables (such as noise or other distractions) or verify if the participants completed the questionnaire accurately. These potential biases due to the online survey indicate the need for some caution in interpreting the results. Our sample is not representative of fathers, because only 5.2% of the enrolled parents were males. Moreover, most participants had a median/high education, and this could represent a sample bias, as parents with lower education and incomes could have had worse outcomes. Finally, although the study confirmed a correlation between children’s behavior and parents’ well-being, we did not properly address the effects of confinement on parent–child relationships. Future surveys should address these important concerns. 

## 5. Conclusions

In conclusion, our study pointed out that the COVID-19 pandemic had a negative impact on the psychological well-being of both children and parents who used DL. This suggests that, although DL could be a valid alternative tool, face-to-face teaching is fundamental, especially at a young age. Indeed, differently from DL, normal teaching encourages dialogue, involvement, and human contact, and builds a better environment in which children may train their skills, including the soft ones.

## Figures and Tables

**Table 1:** Descriptive analysis of children’s and caregivers’ characteristics.

Children	
Age	11.81 ± 3.23
Gender	
Male	47 (48.9%)
Female	49 (51.1%)
**Caregivers**	
Age (years)	44.62 ± 5.30
Gender	
Male	5 (5.2%)
Female	91 (94.8%)
Professions	
Freelancer	22 (22.9%)
Employee	23 (24.0%)
Housewife	10 (10.4%)
Doctor	9 (9.4%)
Healthcare staff	9 (9.4%)
Teacher	10 (10.4%)
Unemployed	6 (6.2%)
	7 (7.3%)

Mean ± standard deviation are used to describe continuous variables; proportions (numbers and percentages) are used to describe categorical variables.

**Table 2:** Average of the clinical scale of caregivers.

Test/Scale	Mean ± SD	Cut-Off
DASS-21 S	**10.66 ± 4.3**	>10
DASS-21 A	**9.03 ± 4.1**	>6
DASS-21 D	8.60 ± 4.7	>10
SDC Q	**4.41 ± 4.1**	>2
SUS Parents	**70.47 ± 19.9**	<50
CDI	**24.04 ± 3.3**	>15
S.T.A.I.C. 1	**41.81 ± 4.9**	>40
S.T.A.I.C. 2	14.23 ± 9.3	>40
SUS Children	**70.46 ± 19.9**	<50

Legend: DASS-21 S = Depression Anxiety Stress Scales—Stress; DASS-21 A = Depression Anxiety Stress Scales—Anxiety; DASS-21 D = Depression Anxiety Stress Scales—Depression; SDC Q= Stress for Distance learning in COVID-19 era Questionnaire; SUS Parents = System Usability Scale; CDI = Children Depression Inventory; S.T.A.I.C. 1 = State-Trait Anxiety Inventory for Children—State; S.T.A.I.C. 2 = State-Trait Anxiety Inventory for Children—Trait; SUS Children = System Usability Scale. Significant mean ± standard deviations are in bold.

## Data Availability

Data could be available on demand to the corresponding author.
